# Investigating the potential antibacterial, anti-biofilm, wound healing and anti-inflammatory activity of the extract of *Aspergillus niger* endophyte isolated from cucumber leaves: in vitro and in vivo study

**DOI:** 10.1186/s12866-025-04134-w

**Published:** 2025-07-07

**Authors:** Maisra M. El-Bouseary, Duaa Eliwa, Mahmoud H. Farghali, Amany E. Ragab

**Affiliations:** 1 Department of Microbiology and Immunology, Faculty of Pharmacy, Tanta University, Tanta, Egypt; 2 Department of Pharmacognosy, Faculty of Pharmacy, Tanta University, Tanta, Egypt

**Keywords:** *Aspergillus niger*, Endophytic fungi, *Cucumis sativus*, LC-HRMS/MS, Anti-inflammatory, Antibacterial, Anti-biofilm, Wound healing

## Abstract

**Background:**

Endophytic fungi are a vast inventory of bioactive compounds, offering potent, cost-effective, renewable, and low-toxicity alternatives for therapeutic applications. The current investigation focused on the endophytic fungus *Aspergillus niger*, which was isolated for the first time from *Cucumis sativus* (cucumber) leaves and subjected to comprehensive evaluation, including anti-inflammatory, antibacterial, anti-biofilm, and in vitro wound healing potential. 18 S rRNA gene sequencing was utilized to identify *A. niger* after isolation, and the fungus was cultivated on Asian rice to produce fungal metabolites. The high-resolution liquid chromatography-mass spectrometry (LC-HRESI-MS/MS) was then used to elucidate its phytochemical profile.

**Results:**

Fingerprint compounds detected in the ethyl acetate of the endophyte *A. niger* (ANM) revealed 15 compounds that are mainly pyrones and quinones in nature, including citric acid, nigerasperone A, aspernigrin A, aspinonene, campyrone B, aurasperone F, and plastoquinone-3. The ANM showed a strong antibacterial activity against *S. aureus* clinical isolates (MIC values ranging from 32 to 512 µg/mL) and a significant reduction in biofilm formation, where the total number of biofilm producers, *S. aureus* isolates, decreased from 19 to 6 after treatment with ½ MIC of ANM. Furthermore, ANM-treated WI38 human fibroblast cells displayed a wound closure percentage of 99.68% ± 0.02 compared to 83.37% ± 0.05 for the control cells. Additionally, the ANM demonstrated potential in promoting wound healing, particularly in infected wounds, through its antimicrobial, anti-inflammatory, and tissue-regenerating properties.

**Conclusions:**

These findings highlight *A. niger* as a valuable source of natural therapeutics. Additional research is needed to explore its key active components and potential side effects.

**Supplementary Information:**

The online version contains supplementary material available at 10.1186/s12866-025-04134-w.

## Introduction

Host-associated fungal communities known as endophytic fungi develop in the tissues of their host plants and generate a diverse range of secondary metabolites with various bioactive characteristics [1]. These fungal-derived compounds have emerged as valuable resources for drug discovery, demonstrating potential for developing novel pharmaceuticals [2, 3]. These endophytes produce variable bioactive compounds which have antimicrobial, antioxidant, anticancer, and anti-inflammatory activities [4–8]. Remarkably, the endophytic fungal secondary metabolites are of exceptional commercial value, particularly biotransformed metabolites with improved bioactivity and bioavailability for the pharmaceutical industry and agrochemical industry [9–11]. The pharmaceutical industry employs fungal biotransformation to optimize drug precursors and reduce synthetic steps for complying with green chemistry principles [12]. There are, however, environmental challenges pending, including metabolic imbalance in scale-up and unanticipated ecologic consequences of fungal dispersion [13]. Biotransformation activities are associated with biosafety concerns such as toxin accumulation (aflatoxin-like compound) and allergenicity from the handling of fungal biomass [11]. Regulatory inadequacies in terms of strain modification and waste treatment present enormous challenges for industrialization [12]. Refinement in CRISPR-guided metabolic engineering and closed bioreactor systems can potentially mitigate these risks [13]. It is crucial that metabolic profiling and strategic strain selection are essential to achieve a balance between safety and therapeutic efficacy [11].

*Cucumis sativus* L., also known as cucumber, ranks as the fourth most cultivated vegetable globally and belongs to the *Cucurbitaceae* family [14]. It has spread across the globe from South Asia and is of immense agricultural significance. Different parts of the cucumber plant, including seeds, fruit pulp, leaves, and peels, have been utilized in the practice of traditional medicine, especially Ayurvedic and Unani medicine, owing to their anti-inflammatory, fever-reducing, and diuretic actions [15, 16]. *C. sativus* is demonstrated to possess antimicrobial, antioxidant, and cytoprotective properties that can be due to its varied phytochemical constituents such as flavonoids, tannins, and saponins [17, 18]. Because of its medicinal importance and likely bioactive richness, *C. sativus* is an excellent candidate for endophytic microorganism isolation, which is likely to play a role in augmenting its therapeutic potential. This formed the basis for selecting *C. sativus* as the source plant in our study.

One of the common endophytic fungi with a variety of identified biologically active compounds is the *Aspergillus* genus [19]. Recently, researchers have become interested in endophytic *Aspergillus sp.* as a promising candidate due to its ability to produce antimicrobial compounds, which makes it perfect for developing novel antimicrobial chemicals. Because of their secondary metabolites, strains of *A. niger* have drawn a lot of attention and are now among the most studied fungal species [20, 21].

*Staphylococcus aureus*, a Gram-positive pathogen and human commensal, colonizes animals and environmental surfaces [22–24]. It causes wound infections, impetigo, folliculitis, as well as systemic conditions like bacteremia, endocarditis, osteomyelitis, sepsis, and toxic shock syndrome [22, 24–27]. *S. aureus* rapidly develops antibiotic resistance via horizontal gene transfer [28]. Infection with methicillin-resistant *S. aureus* (MRSA) is associated with longer hospital stays and higher death rates, compared to the methicillin-sensitive *S. aureus* [29, 30]. In addition to the emergence of MRSA which shows resistance to almost all beta-lactam class of antibiotics, strains resistant to vancomycin, which has long been the antibiotic used to treat MRSA infections, and to daptomycin, ceftaroline, and linezolid, which represent alternatives used in case of resistance to vancomycin, were encountered [28, 31, 32]. Infections with MRSA are difficult to treat because of the protective biofilms produced by *S. aureus* and the fast-evolving mechanisms of resistance. These resistance mechanisms keep traditional treatment approaches from working and emphasize the need for new modalities of treatment [33–37].

Some endophytes with anticancer and antibacterial activities from *Anethum graveolens* were isolated and identified by El-Zehery et al.., including the inhibition of the growth of *S. aureus* and the disruption of biofilm [38]. In addition, antibacterial activity against MRSA of fungal endophytes was reported previously [39–41].

In the present study, we isolated the fungal endophyte *A. niger* from *C. sativus* (cucumber) leaves. We aimed to investigate the anti-staphylococcal activity of the isolated endophyte through both in vitro and in vivo approaches. We further examined the isolated endophyte’s anti-inflammatory properties and possible impact on the wound-healing process after identifying the endophytic fungus chemical profile using the LC-HRESI-MS technology. This study represents the first report on the endophytic fungi *A. niger* associated with *C. sativus*.

## Materials and methods

### Plant source of the endophytic *A. niger*

Fresh and healthy cucumber leaf samples were bought from the nearby farm in Tanta city, Al-Gharbia governorate, Egypt. A staff member of Tanta University’s Botany Department in the Faculty of Science identified it. Tanta University’s Pharmacognosy Department kept a voucher specimen (PD-7–22-D3).

### Isolation and purification of the endophyte *A. niger* from cucumber leaves

The cucumber leaf samples were cleaned by rinsing them with tap water, disinfected for 60 s. by soaking them in a 70% ethanol solution, rinsed three or four times with sterile water, and then dried. Then, using a sterile dissection razor, the leaves were cut into slices (2 × 2 cm)and placed onto agar plates with potato dextrose agar (PDA) medium supplemented with 250 mg/L amoxycillin to inhibit the growth of bacteria. When isolating endophytic fungi from cucumber leaves, negative controls are required in order to identify contamination from the slices’ exterior. Thus, surface-sterilized cucumber leaves are pushed onto a petri dish filled with PDA medium, and inoculation is done on a second PDA plate (negative control). For subsequent purification processes that seek to isolate genuine endophytic fungi, the positive plates are not utilized if fungal growth is seen on the negative control dishes. At room temperature, the plates were incubated for two weeks, or until the fungus had grown substantially. By periodically inoculating growing fungus on agar plates with fresh PDA medium, pure strains of *A. niger* were produced [42, 43].

### Molecular identification of the isolated endophytic fungi

The E.Z.N.A.^®^ Fungal DNA Mini Kit (D3390-01, Omega BIO-TEK, USA) was employed to extract and purify total fungal DNA in accordance with manufacturer procedure. The isolated fungus were then characterized using the 18 S rRNA gene for fungi universal ribosomal. The forward primer was: 5´-TCCGTAGGTGAACCTGCGG-3´, and the reverse primer: was 5´-TCCTCCGCTTATTGATATGC-3´. For data analysis, the Gel documentation system (Geldoc-it, UVP, England) was used with Totallab analysis software (Ver. 1.0.1), which can be found at www.totallab.com [44]. To verify the identity of aligned sequences, BLAST analysis was performed on the NCBI website (http://www.ncbi.nlm.nih.gov/website). ClustalW software analysis was used to calculate genetic distances and multialignments using the Pairwise Distance approach (www.ClustalW.com). The sequences of *Aspergillus* isolates that were accessible in the GenBank were also compared to the nucleotide sequences.

### Preparation of the extract *of A. niger*

The preparation of *A. niger* (ANM) ethyl acetate extract was carried out in order to perform biological testing and further characterisation. A small mass of isolated *A. niger* was aseptically transferred into ten pre-sterilized 1-liter conical flasks, each containing 100 g of Asian rice in sterile water (110 mL). The fungus was grown for four weeks at room temperature and in the absence of light in static conditions. Ethyl acetate (3 × 1 L) was used to extract the fungus’ secondary metabolites by sonication at 50 °C for 15 min then filtered and combined. To obtain an ethyl acetate crude extract (100 gm, 10% yield, brown color), the combined ethyl acetate extract was dried using a rotary evaporator set at 45 °C [45].

### Phytochemical investigation of the ANM

For phytochemical analysis, an Agilent Technologies 6530 Q-TOF LC/MS with an autosampler (G7129A), a Quat. Pump (G7104C), and a Column Comp (G7116A) was utilized. The injection volume was 2 µL. The analytes were separated on a Zorbax RP-18 column from Agilent Technologies (150 mm × 3 mm, dp = 2.7 μm), with a flow rate of 3 mL/min. The solvent gradiant consists of water containing 1% formic acid (A) and acetonitrile containing 1% formic acid (B) as follows: 90% A for 0–2 min, linear gradiant to 80% A for 2–10 min, linear gradiant to 20% A for 10–42 min, linear gradiant to 10% A for 42–53 min, linear gradiant to 100% B for 53–60 min. Using ESI in (-) ionization mode and a capillary voltage of 4500 V, mass spectra were generated. The m/z range in which the mass spectra were recorded was 50–3000 m/z. The drying gas flow was 8 L/min, and the gas temperature was 200 °C. The voltages for the skimmer and fragmentator were set at 65 and 130 V, respectively, while the collision energy was 10 V. The pressure at which nebulization occurred was 58 psig.

### In vitro antibacterial activity

In the current study, 20 *S. aureus* clinical isolates that were obtained from the culture collection of the department of Microbiology and Immunology, Faculty of Pharmacy, Tanta University were employed to investigate the ANM antibacterial effect. Using agar disc diffusion technique, the antibacterial activity of ANM against *S. aureus* (ATCC 25923) reference strain was screened, where 1 disc was saturated with ANM (1000 µg/mL), while the other discs represented the positive control containing gentamicin and the negative control containing ethyl acetate [46]. The broth microdilution method was employed to assess the tested isolates’ minimum inhibitory concentrations (MIC) (*n* = 20) in a 96-well micro-titration plate [47]. Briefly, the extract was serially diluted in sterile Mueller-Hinton broth. Using 96-well microtiter plates, each well received a standardized bacterial inoculum, typically 1 × 10⁶ CFU/mL. Turbidity was measured to evaluate bacterial growth following 18–24 h of incubation at 37 °C. The minimum inhibitory concentration (MIC) is interpreted as the lowest extract concentration that inhibited bacterial growth.

### In vitro anti-biofilm activity

The tested isolates’ capacity to form biofilms and the effect of ANM ($\dfrac{1}{2}$, $\dfrac{1}{4}$, and $\dfrac{1}{8}$ MICs) on this ability were assessed using the crystal violet assay, as previously described [48]. In 96-well microtiter plates, bacterial cultures were cultivated with and without sub-MICs of the extract. They were then incubated overnight at 37 °C to promote the formation of biofilms. After carefully washing the wells to get rid of the planktonic cells, the remaining biofilms were preserved and stained with crystal violet (0.1%). In order to dissolve the bound color, 33% (v/v) glacial acetic acid was used, following washing and air drying. The absorbance at 630 nm was then measured. A decrease in absorbance relative to the untreated control suggested that biofilm development was inhibited. According to the measured optical density (OD) and optical density of control (ODc) values at 630 nm, the tested isolates were categorized into: non-producer, where OD ≤ ODc; weak producer, where ODc < OD ≤ 2×ODc; moderate producer, where 2×ODc < OD ≤ 4×ODc; and strong producer, where 4×ODc < OD [49]. The following equation was employed to determine the percentage reduction in biofilm formation [50]:

$$\:\text{P}\text{e}\text{r}\text{c}\text{e}\text{n}\text{t}\text{a}\text{g}\text{e}\:\text{r}\text{e}\text{d}\text{u}\text{c}\text{t}\text{i}\text{o}\text{n}\:\text{i}\text{n}\:\text{b}\text{i}\text{o}\text{f}\text{i}\text{l}\text{m}\:\text{f}\text{o}\text{r}\text{m}\text{a}\text{t}\text{i}\text{o}\text{n}\:=\frac{\text{C}\text{o}\text{n}\text{t}\text{r}\text{o}\text{l}\:\text{O}\text{D}\:630\:\text{n}\text{m}-\text{T}\text{r}\text{e}\text{a}\text{t}\text{e}\text{d}\:\text{O}\text{D}\:630\:\text{n}\text{m}}{\text{C}\text{o}\text{n}\text{t}\text{r}\text{o}\text{l}\:\text{O}\text{D}\:630\:\text{n}\text{m}\:} \times {\:100}$$

### Bacterial morphological changes induced by ANM

Scanning electron microscopy (SEM) was employed to investigate the effect of ANM on the bacterial morphology. *S. aureus* (S8) isolate was plated in a 6-well cell culture plate and allowed to grow overnight in LB broth without and with ANM at $\dfrac{1}{2}$, $\dfrac{1}{4}$, and $\dfrac{1}{8}$ MICs. After washing thrice with PBS, 2.5% glutaraldehyde in PBS buffer (pH 7.4) was used to fix the attached cells for 2 h at ambient temperature, then subsequently, the cells were fixed at 4 °C for 1 h with 1% Osmium tetroxide (OsO_4_) in PBS buffer (pH 7.4). A section of the base of the plate from each well was placed onto the microscope slide, and ethanol was added, subsequently followed by air drying. The slides were inspected using a scanning electron microscope (SEM) (Akashi Seisakusho, Japan) after mounting on metal stubs and gold sputter coating [51].

### In vitro anti-inflammatory activity of ANM

The anti-inflammatory drug piroxicam and different concentrations of ANM dried extract were prepared in DMSO and suspended in serum-free RPMI medium. ANM cytotoxicity was assesed on WI38 human fibroblast cells. WI38 cells were plated in a 96-well plate (3 × 10^3^ cells/well). The cells were incubated with different ANM concentrations and 10 µg/mL piroxicam in CO_2_ incubator for 48 h. The viability of cells was assesed by MTT assay. Lipopolysaccharide (LPS)-stimulated WI38 cells were used to examine the ANM anti-inflammatory activity by investigating the effect of ANM on the gene expression of a pro-inflammatory cytokine, TNF-α [52, 53]. The cells were plated in a 12-well plate (5 × 10^4^ cells/well) with RPMI complete medium. The cells were incubated for 24 h in presence of LPS (20 µg/mL). After incubation, the plate was subjected to centrifugation for 5 min at a speed of 1650 rpm, and the supernatant was removed. Then, the cells were incubated with 1/10 of IC50 of ANM or 10 µg/mL piroxicam for 48 h. The plate was subjected to centrifugation, and RNA was isolated with the RNA isolation kit (iNtRON Biotechnology, Korea) according to the guidelines that the manufacturer has supplied. The SensiFAST cDNA synthesis kit was used (Bioline, London, UK) to convert RNA (1 µg) into cDNA. Quantitative PCR (qPCR) was employed for gene amplification. Ten microliters of SensiFAST SYBR (Bioline, London), one microliter of cDNA, 0.5 µl of 10 pmoles/mL forward primer, and 0.5 µl of 10 pmoles/mL reverse primer (Table 1) made up the reaction mixture. It was finished with 20 µl of nuclease-free water. CFX96™ Real-Time System (BIO-RAD, USA) was employed to apply the following program: heating the reaction mixture at 95 ºC for 10 min for initial denaturation, followed by 40 cycles of 95 ºC for 15 s for denaturation, 60 ºC for 30 s for annealing and 72 ºC for 30 s for extension. The cycle threshold (Ct) of the housekeeping gene, beta-actin, was used to normalize Ct of TNF-α gene.

**Table 1:** TNF-α and beta-actin primers [54]

Gene	Primer
TNF-α	F- CTCTTCTGCCTGCTGCACTTTG
	R- ATGGGCTACAGGCTTGTCACTC
Beta-actin	F- CACCATTGGCAATGAGCGGTTC
	R- AGGTCTTTGCGGATGTCCACGT

### In vitro wound healing assay

The In vitro wound healing assay was carried out as previously reported [55], with modifications. The human fibroblast cells WI38 (ATCC: CCL-75) were plated in a 24-well plate (10^4^ cells/well) and incubated for 24 h. Following the incubation, the cell monolayer was washed with serum-free RPMI, then scratched with a sterile 200 µL-pipette tip. PBS washing was employed to get rid of the cell debris. The plate was then incubated with or without 1/10 of IC_50_ of ANM for 48 h. The cells migrating in the denuded zone were photographed with phase contrast microscopy. The relative wound size at 0, 24, and 48 h post-wound induction was quantified with the Image J version 1.49 software.

### In vivo wound infection model

#### Animals

Fifteen male Wistar albino rats were obtained from the animal house sited at the Faculty of Veterinary Medicine, Cairo University, Egypt. During a two-week period of acclimatization, the rats were fed standard pelleted food and pure water at 25 ± 2 °C with 12-hour light/dark cycles. All standard procedures of handling of laboratory animals were followed according to ARRIVE guidelines. The in vivo experiment and protocol were endorsed by the research ethical committee of the Faculty of Pharmacy, Tanta University, Egypt (TP/RE/2/25 p-04).

#### Wound infection model

The rats were given a mixture of anesthesia via intraperitoneal injections of ketamine and xylazine (87.5 mg/kg and 12.5 mg/kg, respectively). Following that, a sharp blade was used to shave the backs of rats, and their dorsal skin was wiped with 70% alcohol. The skin biopsy punch was employed to induce approximately 10 mm full-thickness excisional skin incisions in the dorsal skin. The rats were divided randomly into five groups of three each (*n* = 3). The induction of wound infection was performed using 10 µL of the bacterial suspension (10^8^ CFU) in all groups except group I, which represented the negative control (non-infected and nontreated) group [56, 57]. After 30 min of bacterial inoculation, groups I, II, III, IV, and V received vehicle (DMSO: Saline 1:1, 0.25 ml, IP), vehicle (DMSO: Saline 1:1, 0.25 ml, IP), ANM (100 mg/kg, IP), ANM (50 mg/kg, IP), and gentamicin (1 mg/mL, IP), respectively. After 24 h of induction of infection, each wound was examined for signs of infection to confirm the bacterial colonization.

Over the course of the experiment, the treatment that was administered on day 0 was repeated on day 1, 3, and 5. In accordance with the American Veterinary Medical Association’s (AVMA) Guidelines for the Euthanasia of Animals (2020 Edition), rats were anesthetized on the sixth day of the experiment using isoflurane and then euthanized by cervical dislocation.

#### Macroscopic wound contractions

The contractions of the wounds were examined on days 2, 4, and 6 post-wound establishment [57]. To follow up on the wound healing process, images of the wound were captured.

The image analyzer software (Image J.2.0 software, USA) was employed to determine the wound size. The following equation was used to calculate the wound area as a percentage relative to the initial wound size:

$$\:\text{P}\text{e}\text{r}\text{c}\text{e}\text{n}\text{t}\text{a}\text{g}\text{e}\:\text{o}\text{f}\:\text{w}\text{o}\text{u}\text{n}\text{d}\:\text{a}\text{r}\text{e}\text{a}=\frac{\text{W}\text{o}\text{u}\text{n}\text{d}\:\text{s}\text{i}\text{z}\text{e}\:\text{a}\text{t}\:\text{t}\text{h}\text{e}\:\text{t}\text{i}\text{m}\text{e}\:\text{o}\text{f}\:\text{t}\text{a}\text{k}\text{i}\text{n}\text{g}\:\text{t}\text{h}\text{e}\:\text{i}\text{m}\text{a}\text{g}\text{e}}{\text{I}\text{n}\text{i}\text{t}\text{i}\text{a}\text{l}\:\text{w}\text{o}\text{u}\text{n}\text{d}\:\text{s}\text{i}\text{z}\text{e}} \times 100$$

#### Histological examination

After euthanasia, the entire wound, with a margin of approximately 10 mm of surrounding intact skin, was surgically removed for histological analysis. The collected tissues were preserved in 10% neutral formalin. Following its removal from formalin, the tissue was embedded in paraffin wax and sectioned into five-micrometer blocks prior to hematoxylin and eosin (H&E) staining [58].

### Statistical analysis

Graphpad Prism 10 was used to perform the ANOVA test. The data represented the mean ± standard deviation. For the wound healing assay, an unpaired *t*-test was employed, and the data represented the mean ± standard deviation.

## Results

### Identification of the isolated endophytic fungi

**Fig. 1:**
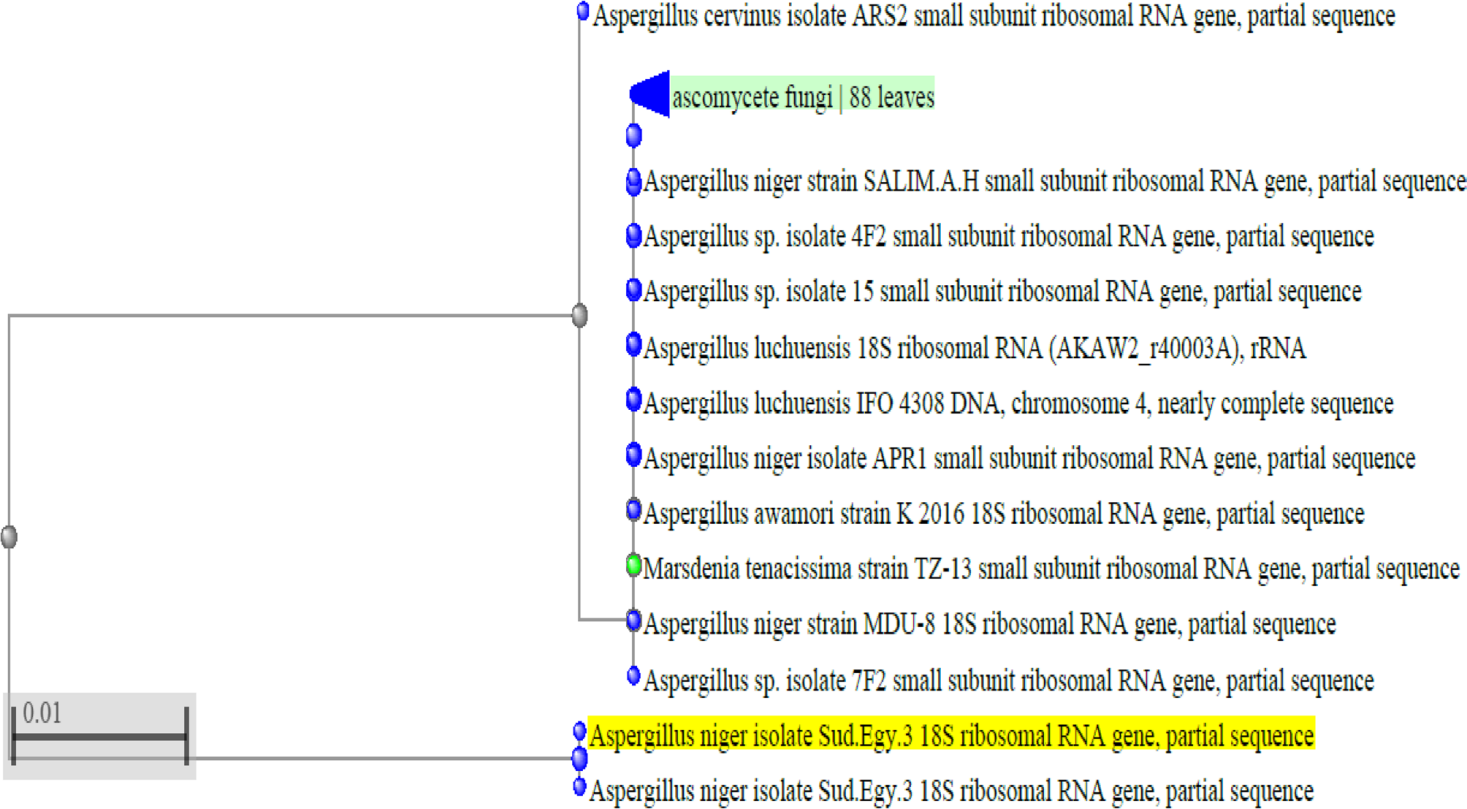
Phylogenetic tree based on the 18 S rRNA gene sequence of the isolated endophytic fungus. Where the isolated strain, *A. niger* (accession number FJ981625.1), is indicated by the yellow highlight

The isolated endophytic fungus was identified as *A. niger* based on its macroscopic and microscopic characteristics as well as the findings of the 18 S rRNA gene sequencing, as shown in Fig. 1 and Supplementary Figure S1. According to Table 2, the accession number for the DNA sequence was FJ981625.1.

**Table 2:** Identification data of *A. niger* by 18 S rRNA sequencing

Identification	Highly similarity Isolates	Highly similarity isolates Accession number	Identity %
*Aspergillus niger*	*Aspergillus niger* strain isolate *C. sativus* leaf small subunit ribosomal 18 S ribosomal RNA, partial and complete sequence	FJ981625.1	100

### Phytochemical characterization of ANM

The ANM was analyzed utilizing HR-LCMS-QTOF technique. The compounds were tentatively identified based on their pseudomolecular ions m/z, MS/MS fragments, free databases search and published literature [59–61].

**Fig. 2:**
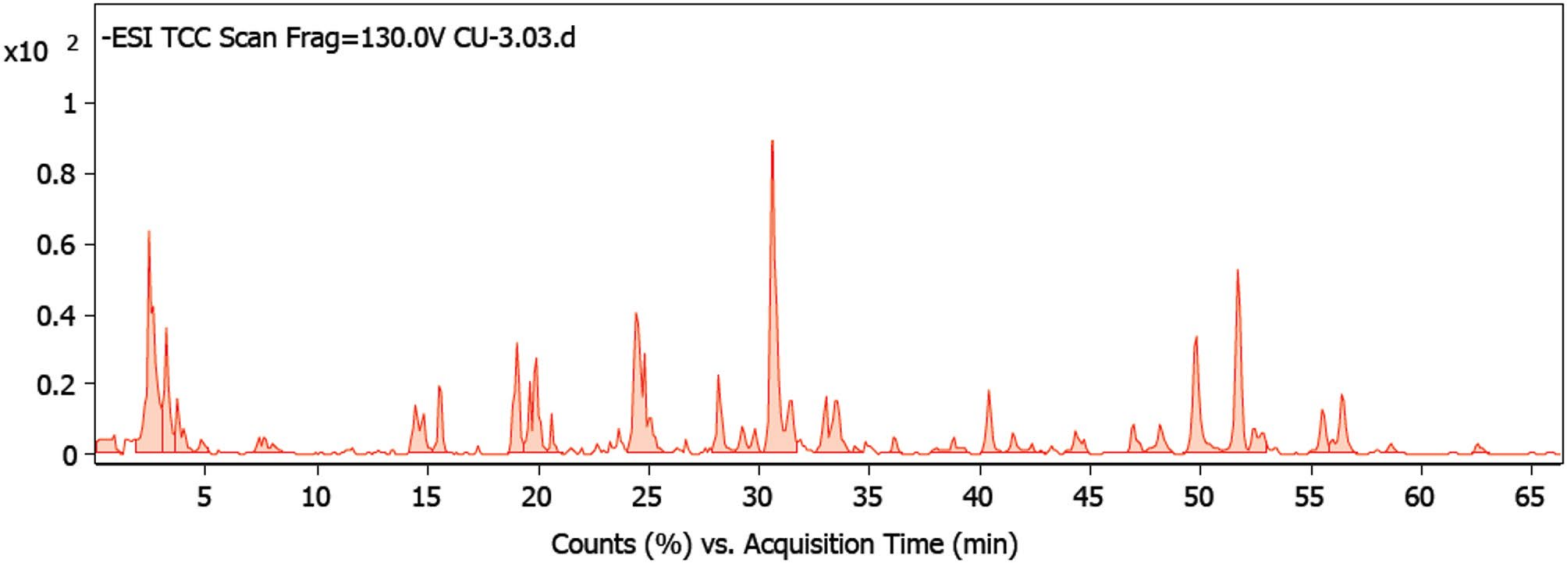
Total ion chromatogram of HR-HPLC-ESI-MS analysis of the ethyl acetate extract of *A. niger* endophyte isolated from cucumber leaves

**Fig. 3:**
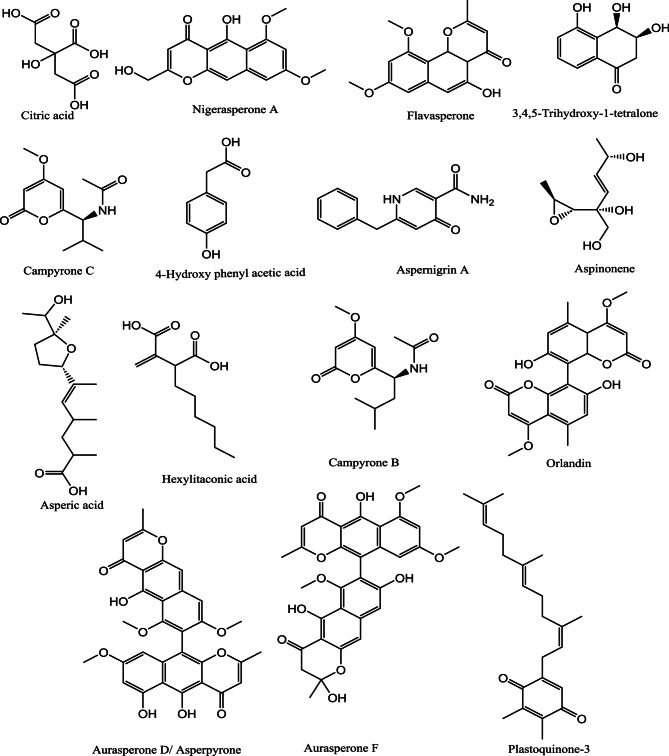
The chemical structures of the compounds identified by HR-HPLC-ESI-MS analysis of the ethyl acetate extract of *A. niger* endophyte isolated from cucumber leaves

The results (Figs. 2 and 3; Table 3) revealed 15 compounds which are mainly pyrones and quinones in nature.

**Table 3:** Fingerprint compounds of *Aspergillus* spp. Detected in the Ethyl acetate of the endophyte *A. niger* isolated from cucumber leaves

No.	Rt (min)	m/z	Molecular weight	Calculated molecular weight	Formula	Identification
1	2.60	191.0199	192.0272	192.027005	C_6_H_8_O_7_	Citric acid
2	3.19	301.0719	302.0793	302.07904	C_16_H_14_O_6_	Nigerasperone A
3	4.96	285.0732	286.0803	286.0841	C_16_H_14_O_5_	Flavasperone
4	12.34	193.0507	194.0579	194.05791	C_10_H_10_O_4_	3,4,5-Trihydroxy-1-tetralone
5	13.00	151.0401	152.0473	152.047345	C_8_H_8_O_3_	4-Hydroxy phenyl acetic acid
6	14.80	227.0837	228.0910	228.089878	C_13_H_12_N_2_O_2_	Aspernigrin A
7	15.54	284.1149	239.1166	239.115759	C_12_H_17_NO_4_	Campyrone C
8	17.90	187.0976	188.1048	188.10486	C_9_H_16_O_4_	Aspinonene
9	19.05	289.1304	253.1321	253.131409	C_12_H_17_NO_4_	Campyrone B
10	21.95	409.0931	410.1004	410.10017	C_22_H_18_O_8_	Orlandin
11	27.40	213.1141	214.1214	214.12051	C_11_H_18_O_4_	Hexylitaconic acid
12	28.21	283.1919	284.1992	284.19876	C_16_H_28_O_4_	Asperic acid
13	29.69	573.1362	574.1435	574.147515	C_31_H_26_O_11_	Aurasperone F
14	31.42	555.1305	556.1377	556.13695	C_31_H_24_O_10_	Aurasperone D/Asperpyrone
15	49.77	339.2339	340.2412	340.24023	C_23_H_32_O_2_	Plastoquinone-3

### In vitro antibacterial activity

Employing the agar-well diffusion method, ANM showed strong antibacterial activity against the reference strain of *S. aureus* (ATCC 25923) (Table 4).

**Table 4:** Inhibition zone diameters (IZDs) of ANM against different bacterial strains

Bacterial reference strain	Inhibition zone diameter (mm)		
	ANM	Gentamicin	DMSO
*S. aureus* (ATCC 25923)	16.67 ± 0.58 ^*^	17.67 ± 0.58 ^*^	10 ± 0.00
*Enterococcus faecalis* (ATCC 29212)	11 ± 1.00 ^#^	15.67 ± 0.58 ^*^	10 ± 0.00
*P. aeruginosa* (ATCC 27853)	10.67 ± 0.58 ^#^	20.33 ± 0.58 ^*^	10 ± 0.00
*K. pneumoniae* (ATCC 700603)	10.33 ± 0.58 ^#^	19 ± 1.00 ^*^	10 ± 0.00
*E. coli* (ATCC 25922)	11 ± 1.00 ^#^	21 ± 1.00 ^*^	10 ± 0.00

Data were recorded as the mean ± SD (*n* = 3). ^*^ Significant vs. DMSO, ^#^ significant vs. *gentamicin*. Each group differed significantly from the others at *p* ≤ 0.05

To determine the MIC values of ANM against clinical isolates of *S. aureus* (*n* = 20), we used the broth microdilution methodology. MIC values were reported to range from 32 to 512 µg/mL (Table 5).

**Table 5:** MIC values of ANM against *S. aureus* clinical isolates (*n* = 20)

MIC (µg/mL)	Number of isolates (%)
32	1 (5)
64	3 (15)
128	9 (45)
256	5 (25)
512	2 (10)

### In vitro anti-biofilm activity

After being treated with $\dfrac{1}{2}$, $\dfrac{1}{4}$, and $\dfrac{1}{8}$ MICs of ANM, the total number of *S. aureus* isolates that produced biofilm decreased from 19 to 6, 9, and 13 (see Supplementary Table S1). Following ANM treatment at $\dfrac{1}{2}$, $\dfrac{1}{4}$, and $\dfrac{1}{8}$ MICs, the Strong biofilm-forming isolates decreased from 40 to 5%, 10%, and 15%, respectively (Table 6).

**Table 6:** Effect of the treatment by sub-MICs of ANM on the biofilm formation

Categories of Biofilm Production	Number of isolates (%)			
	Pre-treatment	Post-treatment		
		$\dfrac{1}{8}$ MIC	$\dfrac{1}{4}$ MIC	$\dfrac{1}{2}$ MIC
None	1 (5)	7 (35)	11 (55)	14 (70)
Weak	4 (20)	6 (30)	4 (20)	3 (15)
Moderate	7 (35)	4 (20)	3 (15)	2 (10)
Strong	8 (40)	3 (15)	2 (10)	1 (5)
Total producers	19 (95)	13 (65)	9 (45)	6 (30)

### Bacterial morphological changes induced by ANM

**Fig. 4:**
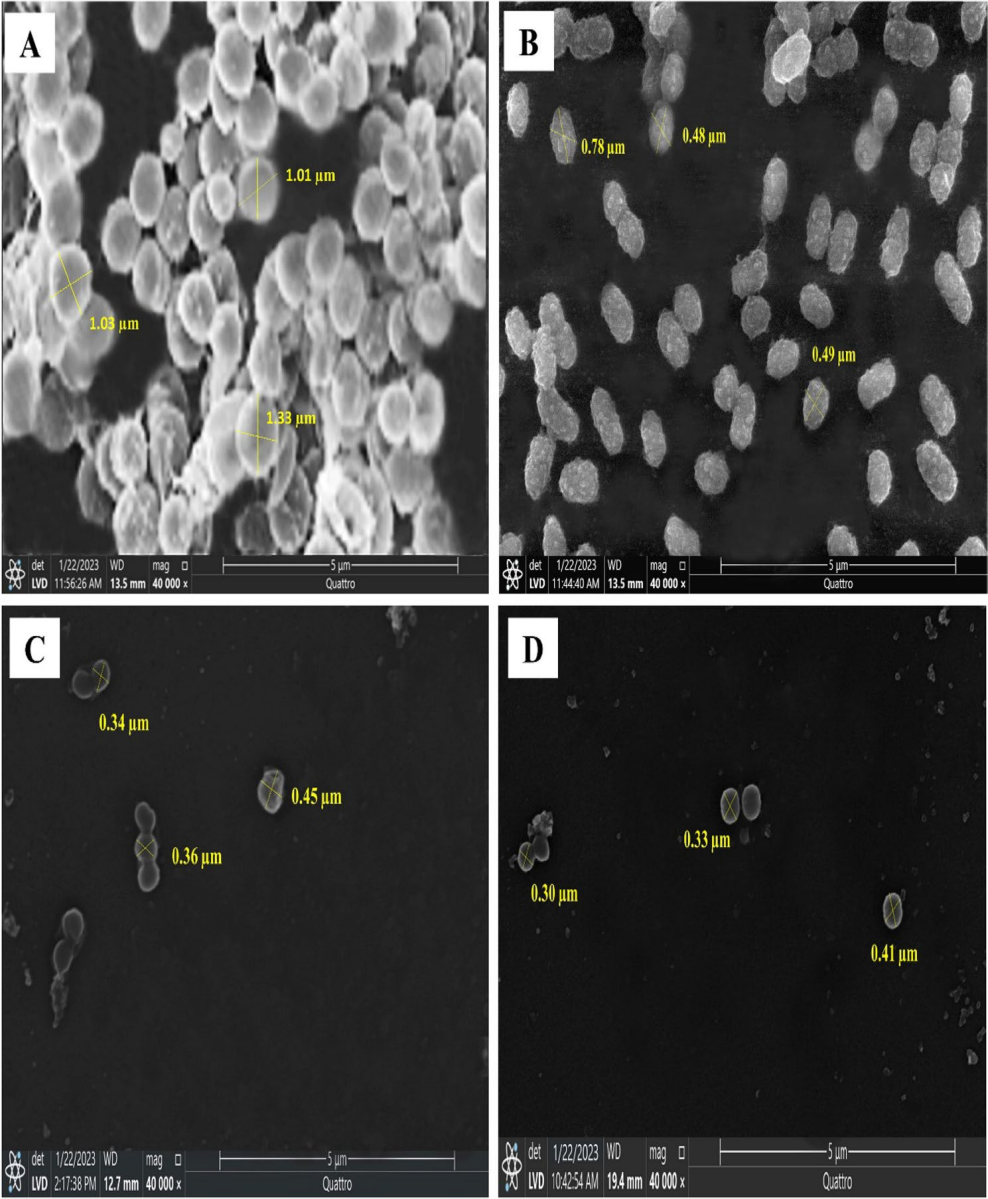
Scanning electron micrograph of *S. aureus* (S8) untreated cells (control) (**A**) and the treated cells with ANM at $\dfrac{1}{8}$ MIC (**B**), $\dfrac{1}{4}$ MIC (**C**), and $\dfrac{1}{2}$ MIC (**D**)

**Fig. 5:**
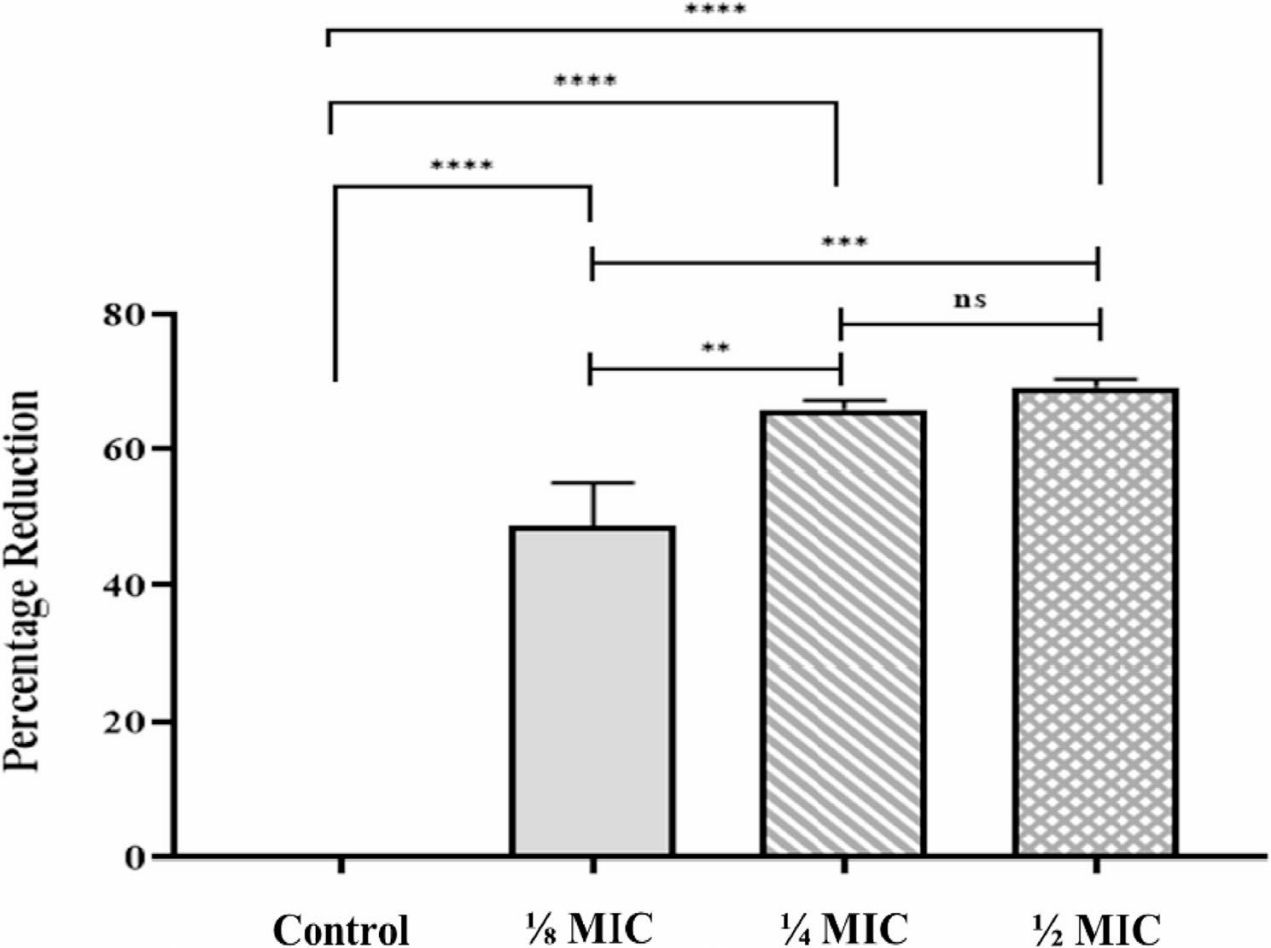
The percentage reduction of *S. aureus* (S8) cell length following the treatment with different sub-MICs of ANM. * *p*-value < 0.05

Sub-MICs of ANM significantly reduce the size of *S. aureus* (S8) cells, distort their shape, and reduce the amount of biofilm matrix that the tested strain forms (Fig. 4). The bacterial cell length was significantly reduced (69%) when treated with a ½ MIC of ANM (*p* < 0.05) (Fig. 5).

### In vitro anti-inflammatory activity

**Fig. 6:**
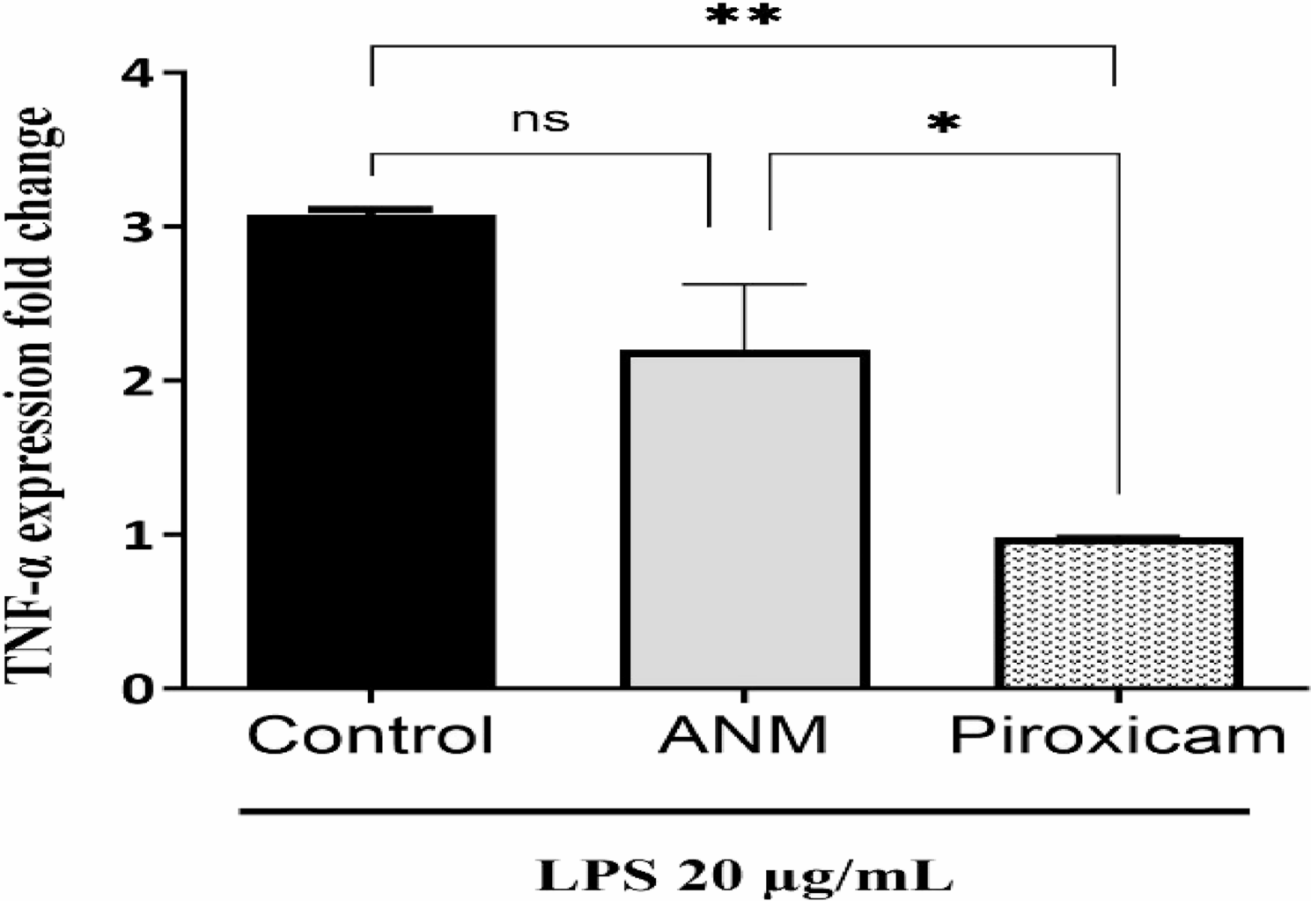
In vitro ANM anti-inflammatory activity. Data represent mean ± standard deviation (*n* = 2), ANOVA test

The cytotoxicity testing revealed that IC_50_ of ANM was 84.05 µg/mL. LPS-stimulated WI38 cells were used to assess the anti-inflammatory activity of 1/10 of IC_50_ of ANM compared to 10 µg/mL piroxicam. The WI38 cells stimulated with LPS showed a marked increase in the expression of TNF-α (~ 3.08-fold ± 0.03). Treatment of the LPS-stimulated cells with ANM resulted in only 2.2-fold increase ± 0.42 in the expression of TNF-α, showing a reduction in TNF-α gene expression, in comparison with the LPS-stimulated control cells. Treatment with piroxicam significantly reduced TNF-α gene expression in LPS-stimulated cells (*p* = 0.007) (Fig. 6).

### Improvement of the in vitro wound healing process

**Fig. 7:**
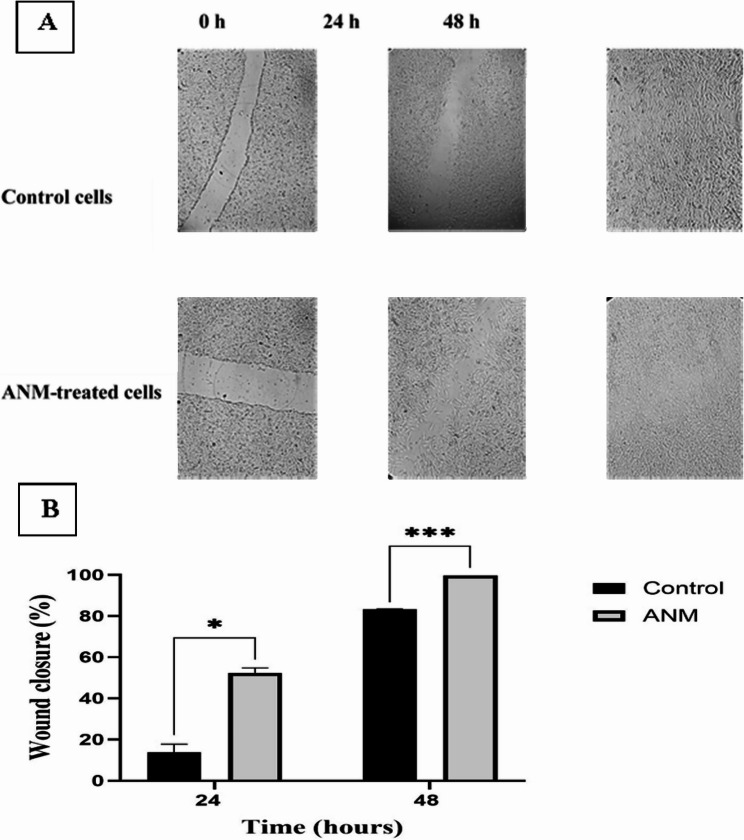
**A** In-vitro wound healing effect of ANM monitored at 0, 24, and 48 h after wound induction (40x magnification). **B** ANM improves the in vitro wound healing process. Data represent mean ± standard deviation (*n* = 2), two-tailed unpaired *t*-test

ANM improved the healing process in WI38 human fibroblast cells (Fig. 7A). As shown in Fig. 7B, the quantification of the relative wound size in control and ANM-treated cells revealed that the percentage of wound closure in ANM-treated cells was remarkably increased (52.37% ± 2.4) compared to the control cells (13.79% ± 3.98) at 24 h after wound induction (*P* = 0.014). At 48 h post-wound induction, ANM-treated cells showed a wound closure percentage of 99.68% ± 0.02 compared to 83.37% ± 0.05 for the control cells (*P* = 0.0005).

### In vivo wound infection model

**Fig. 8:**
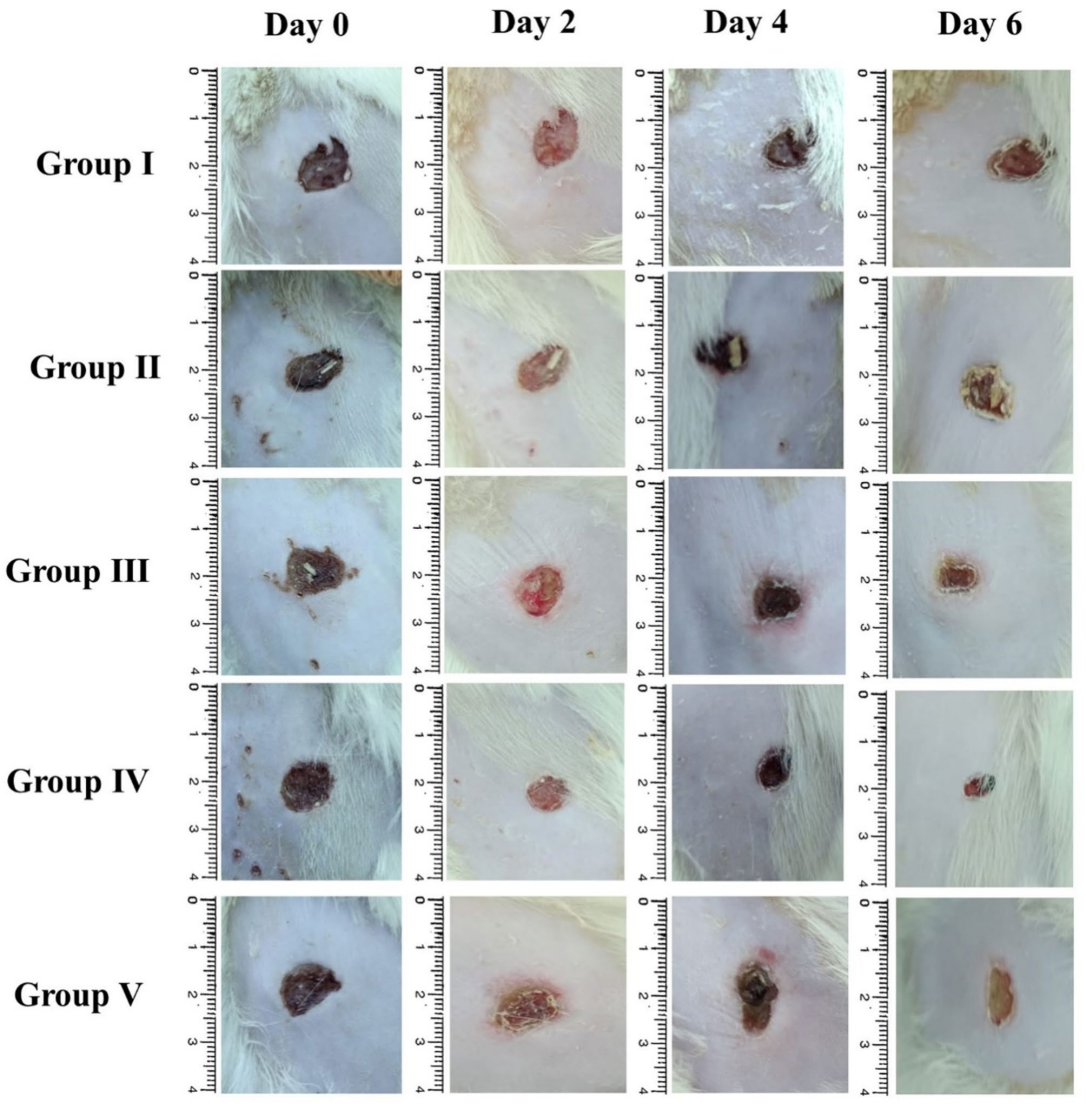
Macroscopic wound contractions were visualized in different animal groups at days 0, 2, 4, and 6

Figure 8 displays the macroscopic wound regeneration rates of the rats under study. On days 0, 2, 4, and 6 of the study, the wound surface size of the group was measured. Wound scabs were observed in group IV (treated with ANM) on day six.

**Fig. 9:**
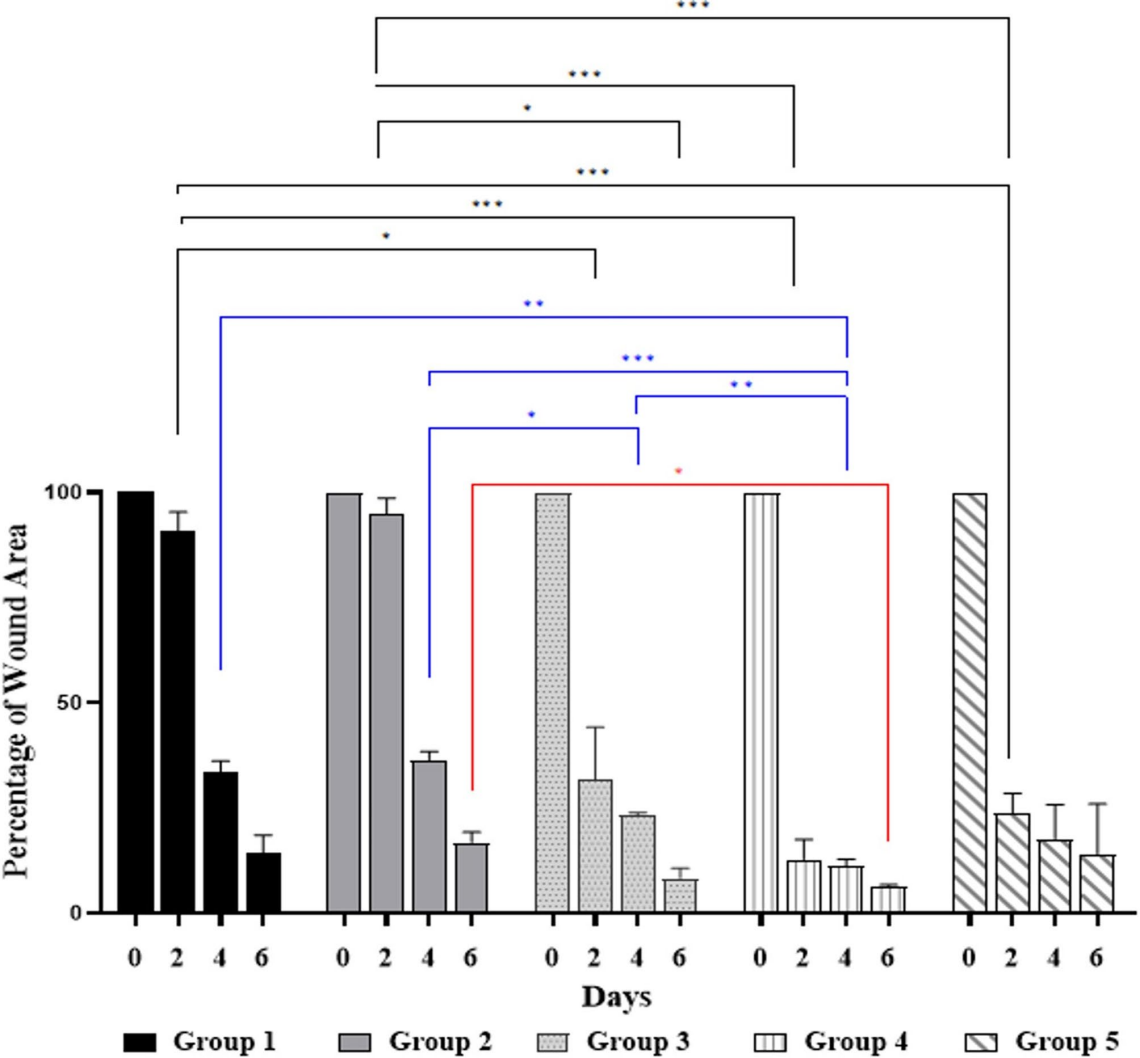
The percentage of wound area for a single rat as a representative recorded in different animal groups at days 0, 2, 4, and 6. * *p*-value < 0.05

**Fig. 10:**
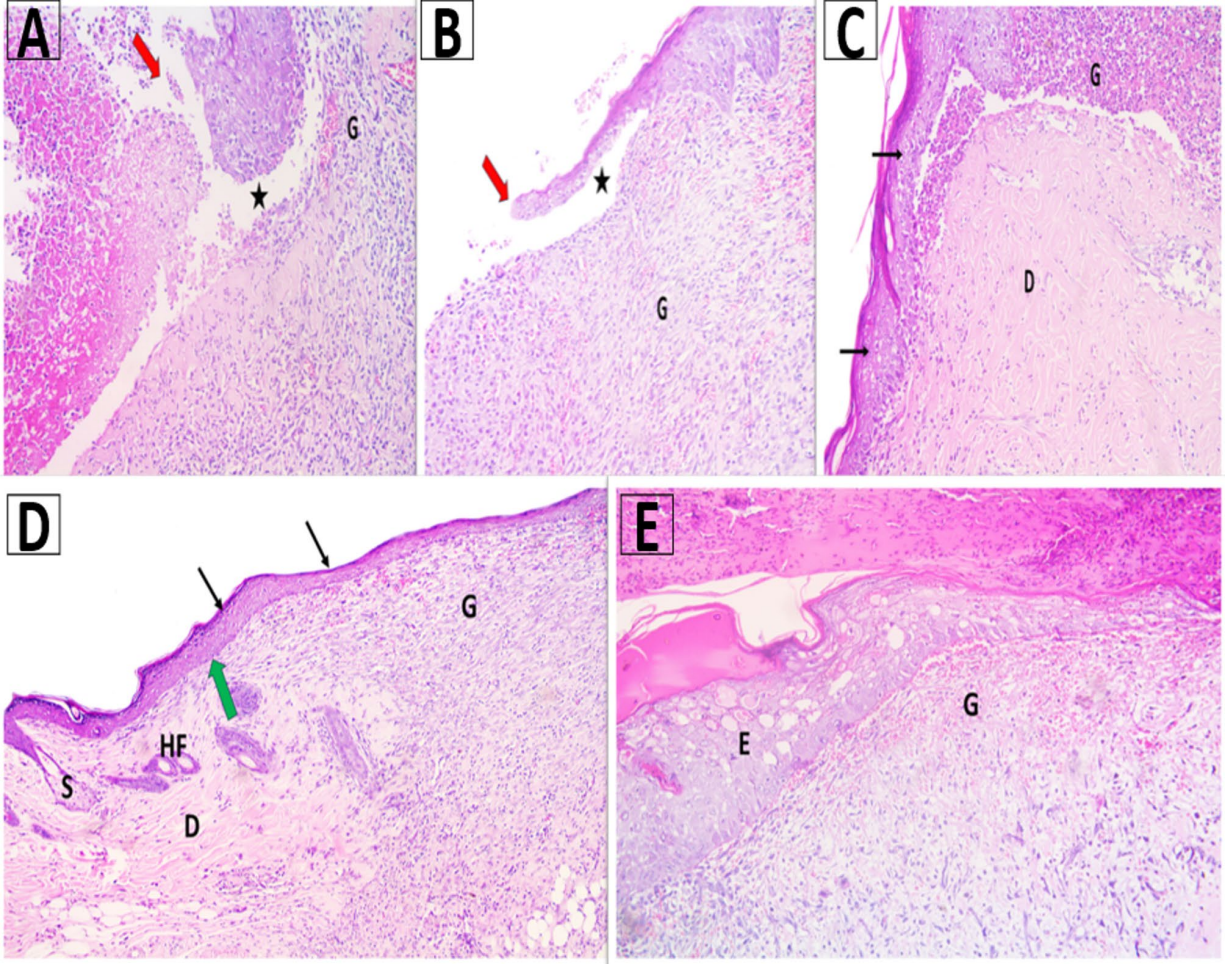
A photomicrograph of skin showing **A** Group I showed disruption of epidermis with separation of it from underlying granulation tissue with absence of skin appendages. **B** Group II revealed disruption of epidermis with separation of it from underlying granulation tissue with absence of skin appendages. **C** Group III showed beginning of re-epithelialization of epidermis, granulation tissue and beginning of organization of connective tissue of dermis. **D** Group IV showed complete re-epithelialization of epidermis (thin arrows), intact basement membrane, organization of connective tissue of dermis, appearance of hair follicle and sebaceous glands. focal area of granulation tissue was seen. **E** Group V showed abnormal arrangement of epidermal cells with cytoplasmic vacuolations (**E**) and granulation tissue (**G**) N.B: disruption of epidermis (red arrows), re-epithelialization of epidermis (thin arrow), separation of epidermis from underlying connective tissue (*), organization of connective tissue of dermis (**D**), granulation tissue (**G**), hair follicle (HF) and sebaceous gland (S). (H&E Mic. Mag. x 200)

The percentage of the wound area is observable in comparison, as represented in Fig. 9. The wound area percentage data shows a consistent trend of wound contraction over time across all groups. On day 2, groups I and II have the highest wound areas (90.84% and 94.82%, respectively), indicating minimal initial healing. In contrast, groups III, IV, and V show significantly lower wound areas (31.82%, 12.77%, and 23.77%, respectively), suggesting a faster response to treatment. By day 4, a notable reduction in wound area is observed across all groups, with group IV exhibiting the lowest wound area (11.27%), followed by group III (23.36%) and group V (17.71%), reinforcing the effectiveness of the treatments with ANM (especially in the treated group receiving a low dose of ANM). By day 6, group IV maintains the smallest wound area (6.35%), demonstrating the most efficient healing process, followed by group III (8.38%) and group V (14.03%). Hematoxylin and eosin (H & E) histological examination of lesion healing across several treated animal groups is illustrated in Fig. 10.

## Discussion

Our findings revealed the antibacterial activity of *Aspergillus niger* against *Staphylococcus aureus*, including multidrug-resistant isolates, with MIC values ranging between 32 and 512 µg/mL. The former observation is attributed to its diverse secondary metabolites, which act synergistically to inhibit bacterial growth. Phenolic compounds, including 3,4,5-trihydroxy-1-tetralone and 4-hydroxyphenyl acetic acid, disrupt bacterial membranes, increase permeability, and produce reactive oxygen species (ROS), which lead to cell destruction and cellular oxidative stress [62]. Organic acids, such as citric acid, create acidic conditions and chelate essential metal ions, disrupting bacterial enzymatic processes [63, 64]. Polyketides, including nigerasperone A, flavasperone, aspernigrin A, aspinonene, campyrone C, campyrone B, orlandin, hexylitaconic acid, asperic acid, aurasperone F, and aurasperone D/asperpyrone, inhibit the bacterial enzymes and bind to DNA to disrupt replication and transcription [65–68].

Earlier research had documented diverse pharmacological activities of *A. niger*-derived bioactive compounds, such as antimicrobial, anticancer, anti-angiogenic, and immunomodulatory [69–71]. Although endophytes in some cases may produce the same or structurally related compounds as their host plant, it does not hold across all cases. In the present research, endophytes collected from *C. sativus* did not produce the same metabolites as reported by the plant in many studies. This difference may be due to variation in the biosynthetic gene clusters, absence of host-specific signaling compounds under artificial cultivation media, or the specialized metabolic potential of the isolated organisms. Nevertheless, the occurrence of unique bioactive compounds suggests that endophytes are great sources of new metabolites, be they host mimic compounds or otherwise.

Our results showed that the ethyl acetate extract of *A. niger* from *Cucumis sativus* leaves improved the in vitro wound healing process in the human fibroblast cells WI38 and had an anti-inflammatory effect indicated by the marked reduction in the expression level of TNF-α in LPS-stimulated WI38. In agreement with our results, it was reported that titanium dioxide nanoparticles biosynthesized by *A. niger* improved the in vitro wound healing process in dermal cells and had an anti-inflammatory effect indicated by the inhibition of human erythrocytes hemolysis [72]. Notably, it was reported that silver nanoparticles produced from an *A. niger* extract had an anti-migration effect on HeLa carcinoma cells, as indicated by the scratch wound healing assay [70]. Baz et al.. reported that *A. niger* culture filtrate extract significantly reduced TNF-α production in MCF-7 cancer cells [71].

Additionally, *A. niger* extract’s biofilm inhibitory activity against *S. aureus* biofilms comes from its capacity to suppress the development of the biofilm, break down mature biofilms, and inhibit the adhesion of bacteria [73]. Secondary metabolites such as phenolic compounds interfere with the integrity of the biofilm matrix by disrupting the extracellular polymeric substances (EPS) that bind the biofilm together, causing the structure to destabilize [74]. Additionally, polyketides impact quorum sensing interference, the quorum sensing mechanism used for bacterial communication to mature and sustain biofilm [74]. This general activity signifies *A. niger* extract presenting an integrated antibacterial activity and a rich source of antibiofilm compounds, which is efficacious in fighting infections caused by *S. aureus* biofilms.

The extract of *A. niger* has been promising the curing of wounds, especially infected wounds, due to its antimicrobial, anti-inflammatory, and tissue-healing properties. Its bioactive metabolites act by inhibiting the bacterial infection by inhibiting the bacterial cell membrane and biofilms, which are usually the prime barriers in wound healing [74]. All these activities make *A. niger* extract a potential drug for infected wound healing.

## Conclusion

The findings of this study underscore the remarkable therapeutic potential of *Aspergillus niger* extract, particularly in combating multidrug-resistant *Staphylococcus aureus* and disrupting biofilms. Its diverse bioactive metabolites exhibit potent antibacterial, anti-inflammatory, and wound-healing properties, making it a valuable natural alternative for combating bacterial infection. The extract’s ability to enhance fibroblast migration, suppress TNF-α expression, and destabilize biofilms highlights its multifaceted role in wound care. These results position *A. niger* as a powerful candidate for novel antimicrobial and wound-healing therapies. The outcomes of our research are the cornerstone of future inquiries on the isolation of bioactive molecules from endophytic fungal extracts, specifically those that are yet to be isolated individually. Research in the future ought to be focused on identifying which of the compounds has the most significant effect. Further study is warranted to evaluate the clinical utility of these extracts in the management of biofilm infections and chronic wounds. Additional anti-inflammatory-guided fractionation is ongoing to purify and identify active compounds in a future study that may serve as promising starting point for developing and discovering new and potent pharmacological agents.

## Supplementary Information

Supplementary Material 1.

## Data Availability

All data generated or analyzed during this study are included in this article and supplementary file.
