# Supplementary material for: Investigating the potential antibacterial, anti-biofilm, wound healing and anti-inflammatory activity of the extract of Aspergillus niger endophyte isolated from cucumber leaves: in vitro and in vivo study
Source: BMC Microbiol. 2025 Jul 7;25:420. doi: 10.1186/s12866-025-04134-w (PMC12232745; doi:10.1186/s12866-025-04134-w)
Supplement: Supplementary file 1 — Supplementary Material 1. [file 12866_2025_4134_MOESM1_ESM.docx]

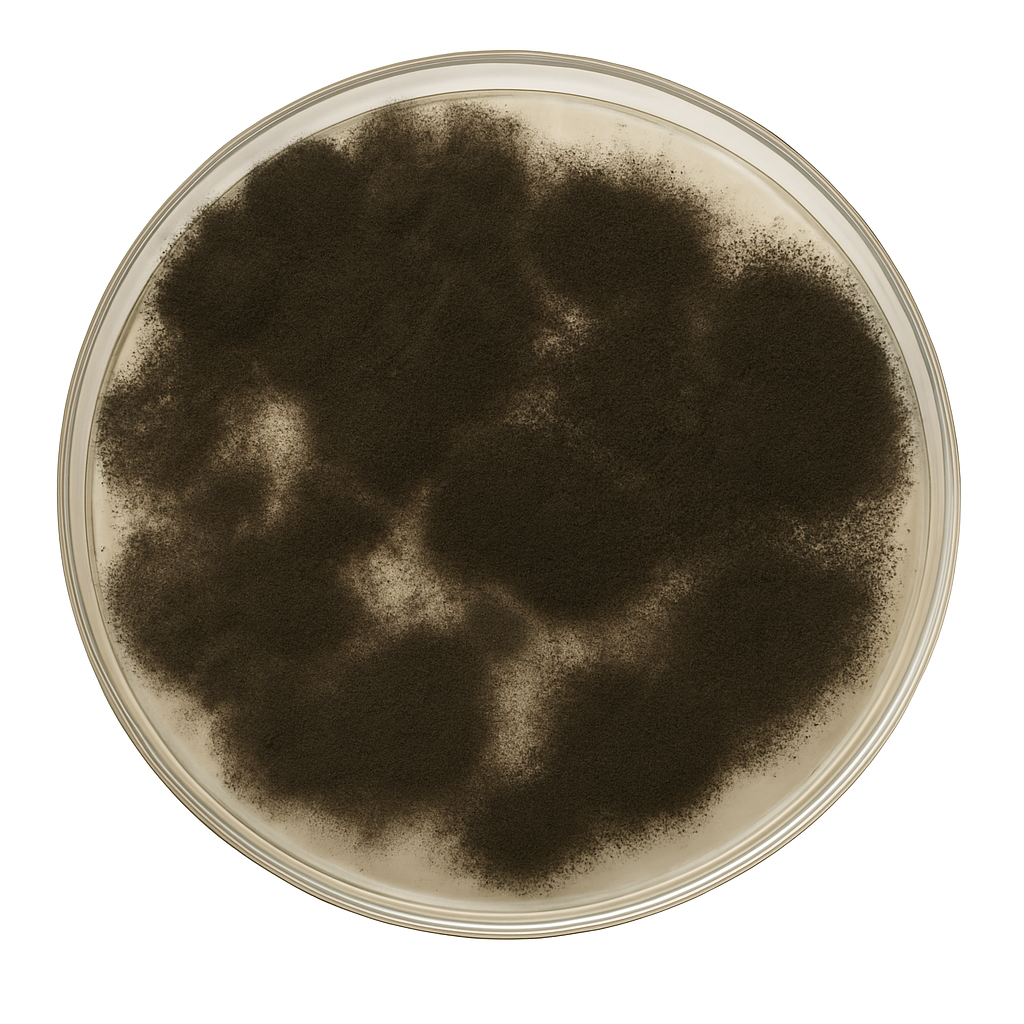


**Figure S1**: The isolated fungal endophyte *Aspergillus niger* culture.

**Table S1**. MIC values of ANM against *S. aureus* clinical isolates and the effect of the treatment by ANM at sub-MICs on the biofilm formation. (S; strong producer, M; moderate producer, W; weak producer, N; non-producer).

| **Isolate code** | **MIC (****µg/ml)** | **Resistance Pattern*** | **Pre-treatment** | **Post-treatment** | | |
| --- | --- | --- | --- | --- | --- | --- |
|  |  |  |  | ⅛ MIC | ¼ MIC | ½ MIC |
| **S1** | 64 | **AZM-GEN-P** | **W** | **N** | **N** | **N** |
| **S2** | 32 | **AZM-CIP-P** | **N** | **N** | **N** | **N** |
| **S3** | 256 | **AZM-CIP-GEN-P** | **S** | **S** | **S** | **M** |
| **S4** | 64 | **AZM-C-P-T** | **W** | **N** | **N** | **N** |
| **S5** | 128 | **CIP-GEN- LEV-P** | **M** | **W** | **W** | **N** |
| **S6** | 256 | **CIP-LEV-LZ-P** | **M** | **W** | **W** | **N** |
| **S7** | 128 | **AZM-CIP-LEV-P-SXT** | **S** | **M** | **M** | **M** |
| **S8** | 128 | **AZM-CIP-LEV-P-RA** | **S** | **M** | **M** | **W** |
| **S9** | 256 | **AZM-FOX-LZ-OX-P** | **M** | **W** | **N** | **N** |
| **S10** | 128 | **AZM-CIP-GEN-LEV-P** | **M** | **W** | **N** | **N** |
| **S11** | 256 | **AZM-C-FOX-OX-P** | **M** | **W** | **N** | **N** |
| **S12** | 128 | **AZM-CIP-LEV-P-T** | **W** | **N** | **N** | **N** |
| **S13** | 64 | **AZM-C-CIP-LEV-P** | **M** | **N** | **N** | **N** |
| **S14** | 128 | **AZM-CIP-LEV-P-RA** | **W** | **N** | **N** | **N** |
| **S15** | 512 | **AZM-C-CD-OX-P-T** | **S** | **S** | **S** | **S** |
| **S16** | 256 | **C-CIP-GEN-LEV-P-T** | **S** | **S** | **W** | **W** |
| **S17** | 128 | **AZM-CD-CIP-LZ-P-T** | **S** | **W** | **N** | **N** |
| **S18** | 128 | **AZM-C-CIP-LEV-P-SXT-T** | **M** | **N** | **N** | **N** |
| **S19** | 512 | **C-CIP-FOX-LZ-OX-P-SXT** | **S** | **M** | **M** | **W** |
| **S20** | 128 | **AZM-C-CIP-LEV-P-RA-SXT** | **S** | **M** | **W** | **N** |

* (AZM) azithromycin, (C) chloramphenicol, (CD) clindamycin, (CIP) ciprofloxacin, (FOX) cefoxitin, (GEN) gentamicin, (LEV) levofloxacin, (LZ) linezolid, (OX) oxacillin, (P) penicillin, (SXT) trimethoprim-sulfamethoxazole, (T) tetracycline, (RA) rifampicin.
